# A robust pipeline for ranking carrier frequencies of autosomal recessive and X-linked Mendelian disorders

**DOI:** 10.1038/s41525-022-00344-7

**Published:** 2022-12-19

**Authors:** Wenjuan Zhu, Chen Wang, Nandita Mullapudi, Yanan Cao, Lin Li, Ivan Fai Man Lo, Stephen Kwok-Wing Tsui, Xiao Chen, Yong Lei, Shen Gu

**Affiliations:** 1 Centre for Cardiovascular Genomics & Medicine, Faculty of Medicine, The Chinese University of Hong Kong, Hong Kong SAR, China; 2 Division of Medical Sciences, Department of Medicine & Therapeutics, Faculty of Medicine, The Chinese University of Hong Kong, Hong Kong SAR, China; 3 School of Biomedical Sciences, Faculty of Medicine, The Chinese University of Hong Kong, Hong Kong SAR, China; 4 Bionano Genomics, San Diego, CA 92121 USA; 5 Department of Endocrine and Metabolic Diseases, Shanghai Institute of Endocrine and Metabolic Diseases, National Clinical Research Centre for Metabolic Diseases, Key Laboratory for Endocrine and Metabolic Diseases of the National Health Commission, State Key Laboratory of Medical Genomics, Ruijin Hospital, Shanghai Jiao Tong University School of Medicine, Shanghai, China; 6 National Research Center for Translational Medicine, National Key Scientific Infrastructure for Translational Medicine, Shanghai Jiao Tong University, Shanghai, China; 7 Department of Health, Clinical Genetic Service, Hong Kong SAR, China; 8 Dr. Li Dak Sum-Yip Yio Chin Center for Stem Cells and Regenerative Medicine and Department of Orthopedic Surgery of The Second Affiliated Hospital, Zhejiang University School of Medicine, Hangzhou, China; 9 Key Laboratory of Tissue Engineering and Regenerative Medicine of Zhejiang Province, Zhejiang University School of Medicine, Hangzhou, China; 10 Zhejiang University-University of Edinburgh Institute & School of Basic Medicine, Zhejiang University School of Medicine, Hangzhou, China; 11 Department of Sports Medicine, Zhejiang University School of Medicine, Hangzhou, China; 12 China Orthopedic Regenerative Medicine Group (CORMed), Hangzhou, China; 13 School of Medicine, The Chinese University of Hong Kong (Shenzhen), Shenzhen, China; 14 Key Laboratory for Regenerative Medicine, Ministry of Education, School of Biomedical Sciences, Faculty of Medicine, The Chinese University of Hong Kong, Hong Kong SAR, China; 15 Kunming Institute of Zoology Chinese Academy of Sciences, The Chinese University of Hong Kong Joint Laboratory of Bioresources and Molecular Research of Common Diseases, Hong Kong SAR, China; 16 Hong Kong Branch of CAS Center for Excellence in Animal Evolution and Genetics, The Chinese University of Hong Kong, New Territories, Hong Kong SAR, China

**Keywords:** Disease prevention, Genetics research

## Abstract

Single gene disorders are individually rare but collectively common leading causes of neonatal and pediatric morbidity and mortality. Both parents or the mothers of affected individuals with autosomal recessive or X-linked recessive diseases, respectively, are carrier(s). Carrier frequencies of recessive diseases can vary drastically among different ethnicities. This study established a robust pipeline for estimating and ranking carrier frequencies of all known 2699 recessive genes based on genome-wide sequencing data in healthy individuals. The discovery gnomAD cohort contained sequencing data on 76,156 genomes and 125,748 exomes from individuals with seven ethnicity backgrounds. The three validation cohorts composed of the SG10K Project with 4810 genomes on East Asian and South Asian, the ChinaMAP project with 10,588 Chinese genomes, and the WBBC pilot project with 4480 Chinese genomes. Within each cohort, comprehensive selection criteria for various kinds of deleterious variants were instituted, including known pathogenic variants (Type 1), presumably loss-of-function changes (Type 2), predicted deleterious missense variants (Type 3), and potentially harmful in-frame INDELs (Type 4). Subsequently, carrier frequencies of the 2699 genes were calculated and ranked based on ethnicity-specific carrier rates of Type 1 to Type 4 variants. Comparison of results from different cohorts with similar ethnicity background exhibited high degree of correlation, particularly between the ChinaMAP and the WBBC cohorts (Pearson correlation coefficient *R* = 0.92), confirming the validity of our variant selection criteria and the overall analysis pipeline.

## Introduction

A Mendelian disease is a genetic disorder caused mainly by abnormalities in a single gene or locus in the human genome. The inheritance patterns of these diseases follow a dominant or recessive mode and are either autosomal or X-linked, depending on the chromosomal locations of the affected genes. Mendelian diseases may be individually rare but are collectively common. They are leading causes of neonatal morbidity and mortality, and in recent studies, 36.7–57.0% of critically ill infants were molecularly diagnosed with Mendelian diseases through rapid clinical exome sequencing or whole genome sequencing^1–3^. Specifically, 41.5% of the diagnosed disorders were autosomal recessive (AR) with both parents being carriers of the gene variant, and 6.5% were X-linked recessive (XLR) diseases with a maternal carrier^1^. Except for extremely rare circumstances of uniparental disomy or de novo changes, individuals with AR diseases inherit two pathogenic variants (one from each parent). For XLR diseases, pathogenic variants may be inherited from the mother or arise de novo.

The devastating clinical consequences have led to a longstanding fight to ameliorate and prevent recessive diseases worldwide. Such efforts have been achieved through two major approaches, namely newborn screening (NBS) and prenatal/pre-pregnancy carrier screening. NBS is critical for the early diagnosis and effective management of inborn errors of metabolism (IEM), of which the vast majority are recessive diseases^4^. Prenatal/pre-pregnancy carrier screening uses genetic testing to detect the carrier status of prospective parents, who then can make informed reproductive decisions based on their personal preferences and values in relation to preimplantation genetic diagnosis, in vitro fertilization, or invasive prenatal testing. Advances in tandem mass spectrometry and next generation sequencing (NGS) have enabled the simultaneous detection of many genetic conditions through NBS and carrier screening, respectively. Therefore, expanded NBS screening 30–50 conditions and expanded carrier screening (ECS) detecting dozens to hundreds of recessive diseases are being widely implemented in developed countries.

Carrier frequencies of recessive diseases vary drastically among different ethnic groups^5–7^. For example, cystic fibrosis, which is caused by pathogenic variants in the *CFTR* gene, is the most common life-limiting AR disease affecting Caucasians. The *CFTR* carrier frequency is as high as 1 in 25 in Caucasian and Ashkenazi Jewish populations, but is much lower among Asian-Americans (1 in 94)^8^. In contrast, beta-thalassemia, another common AR disorder caused by deleterious changes in the *HBB* gene, is highly frequent in populations of Mediterranean, African, and Central and Southeast Asian, but is much rarer among those of Caucasian descent^8^. These statistics suggest that recessive gene panels selected for NBS and carrier screening should be based on population-specific carrier frequencies in countries and regions with a single majority ethnicity group.

Previously documented carrier frequencies of relatively large gene panels (*e.g*. more than 100 genes included) were primarily estimated based on genome-wide sequencing data of unaffected individuals^9–11^. Limited reports were available for carrier frequencies of large gene panels in individuals actually subjected to genetic testing^5,6^. Note that both estimation and actual observation of population-specific carrier frequencies were mostly on individuals resided in the United States (US) with self-report ethnicities. In geographic regions without accessible ECS, carrier frequencies of most genes remained unknown. Thus, this study aimed to develop an unbiased and robust pipeline for ranking carrier frequencies of all known recessive genes based on genome-wide sequencing data. Elaborated criteria to select deleterious variants were rigorously established. Comparison of results based on sequencing data from different cohorts with similar ethnicity background exhibited high degree of correlation, confirming the validity of our method. With increasing accumulation of region-specific genome sequencing data, our pipeline is readily applicable to establish ranking of carrier frequencies of the local population, providing integral information on designing territorial NBS and ECS panels.

## Results

### Selection of deleterious variants in recessive genes

In this study, sequencing results from the publicly available Genome Aggregation Database (gnomAD) were extracted and analyzed as the discovery cohort. gnomAD aggregated high-quality, uniformly processed whole genome sequencing (WGS) data from 76,156 individuals and whole exome sequencing (WES) data from 125,748 individuals^12^. These are primarily unaffected individuals from case-control studies of common adult-onset diseases. Particularly, samples from second-degree or more closely related individuals and from individuals as well as their first-degree relatives known to have severe childhood-onset diseases were removed^12^. Therefore, the variant data from gnomAD fulfills the requirement of our analysis on the carrier frequencies of recessive diseases in a generally healthy population. Variant information from gnomAD was extracted and only high-quality variants were retained and annotated (see Methods). Furthermore, gnomAD represents individuals with different self-defined ethnicity background, including African/African American (AFR), Latino/Admixed American (AMR), Ashkenazi Jewish (ASJ), East Asian (EAS), Finnish European (FIN), non-Finnish European (NFE), and South Asian (SAS) (Supplementary Dataset 1). Ethnicity-specific allele frequencies of each variant could be obtained.

**Fig. 1:**
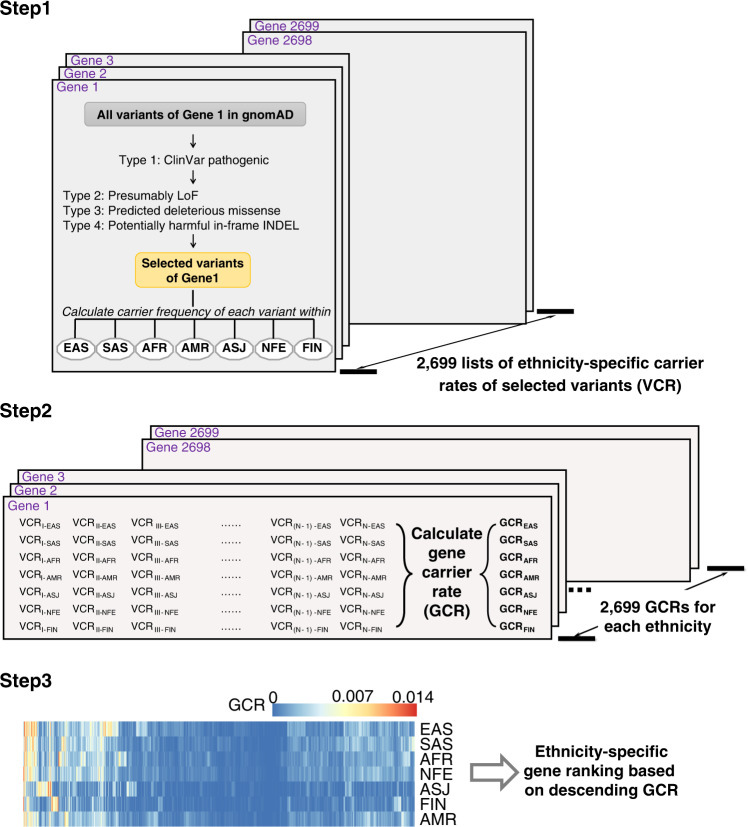
Analysis workflow of ranking carrier frequencies in all recessive genes. Steps in the discovery (gnomAD) cohort are illustrated. EAS East Asian, SAS South Asian, AFR African/African American, AMR Latino/Admixed American, ASJ Ashkenazi Jewish, NFE European (non-Finnish), FIN European (Finnish), VCR variant carrier rate, GCR gene carrier rate.

2699 known disease-causing recessive genes documented in the Online Mendelian Inheritance in Man (OMIM) database were considered in this study, including 2525 autosomal recessive and 174 X-linked genes (Supplementary Dataset 2, see Methods). As shown in the workflow (Fig. 1), high-quality gnomAD variants aligned to the GRCh38 human genome assembly reference in each gene were processed. Overall, 48,198,273 gnomAD variants were found in the 2699 genes. Among these variants, we aimed to select those that either have been reported in affected patients or could potentially induce deleterious effects on gene function. Four categories of variants were retained based on the following criteria:

#### Type 1 variants – known pathogenic changes in ClinVar

ClinVar is a well-established database used to document the pathogenicity of genetic alterations in the context of human diseases^13^. Variants in ClinVar that have been categorized as “pathogenic” were either reported in the medical literature or submitted by clinical diagnostic laboratories following observation in affected individuals. Therefore, ClinVar is a generally trustworthy resource for disease-causing variants in patients.

We have defined 28,017 pathogenic ClinVar variants in our list of 2699 genes as Type 1 variants for downstream calculations (see Methods for detailed selection criteria). ClinVar variant of uncertain significance (VUS) and undocumented variants were further filtered as potentially deleterious Type 2 to Type 4 changes based on the criteria described below. Notably, any variant with a homozygous call in gnomAD (including hemizygous chromosome X variants in males and homozygous chromosome X variants in females) were removed. Further, variants with alternative allele frequencies (AF) ≥ 0.005 were removed following previous studies^5,9^. As a result, 45,354,101 ClinVar VUS and undocumented variants were further analyzed.

#### Type 2 variants – presumed loss-of-function (LoF) changes

Presumed LoF changes include five types of variant consequences designated as HIGH impact alterations in Ensembl: stop gained (nonsense), start lost, frameshift, splice acceptor and splice donor. Notably, if a specific variant results in different consequences in different transcripts due to alternative splicing, the consequence with the most severe impact was considered. Additional filtering was applied to exclude nonsense changes within 50 bp of the final exon junction that could potentially result in an escape of nonsense-mediated decay^14^. In total, 119,452 Type 2 variants were identified (Supplementary Dataset 3).

#### Type 3 variants – predicted deleterious missense changes

**Fig. 2:**
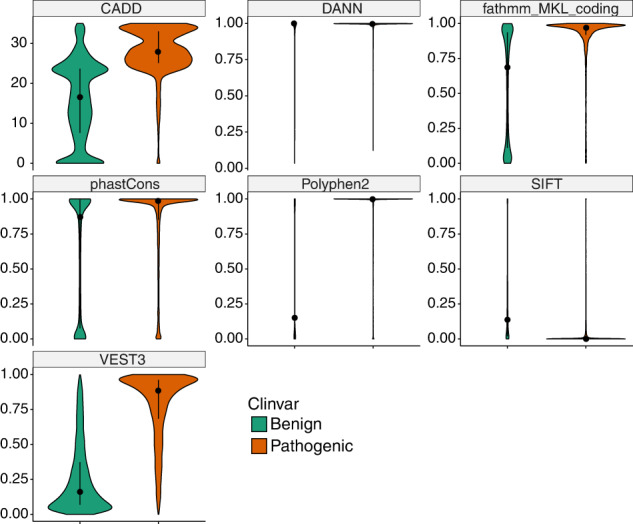
Violin plots comparing scores of the selected seven variant analysis tools. All gnomAD missense variants defined as pathogenic/likely pathogenic and benign/likely benign in ClinVar were evaluated. Violin plots illustrate the median value (dot) and the 25th to 75th percentile range (black line). In each plot, benign variants are on the left and pathogenic variants are on the right. The deleteriousness scores are along the y-axis. Higher values indicated a higher probability that the variant is damaging in all scores except for SIFT, where a low score is associated with deleteriousness. The y-axis for CADD is a logarithmically transformed score, and the rest are linear probabilities. The x-axis represents the probability density of variants along the range of scores. The CADD plot appears different because its y-axis is on a logarithmic instead of linear scale. Calculated mean scores with standard deviations are listed in Supplementary Dataset 4. See also Supplementary Fig. 1.

We obtained prediction scores of missense variant pathogenicity from dbSNFP v3.5a^15^, which included results from fifteen analysis tools (CADD, DANN, FATHMM, GERP, LRT, M-CAP, MetaLR, MetaSVM, MutationAssessor, MutationTaster, Polyphen2, SIFT, VEST3, fathmm_MKL_coding and phastCons). These tools predict whether an amino acid substitution is deleterious based on evolutionary conservation, the amino acid sequence and protein structure, the derived allele versus de novo simulation, *etc*. To select the most informative tools, all gnomAD missense variants categorized as pathogenic (P, 7384 variants), likely pathogenic (LP, 3688 variants), benign (B, 27,759 variants) and likely benign (LB, 17,944 variants) in ClinVar were used to evaluate their performance. Among the fifteen analysis tools, seven tools (CADD, DANN, fathmm_MKL_coding, phastCons, Polyphen2, SIFT and VEST3) clearly distinguished ClinVar pathogenic missense variants from benign missense changes (Fig. 2, Supplementary Fig. 1, Supplementary Dataset 4). Of the seven tools, combination of five tools, namely CADD, DANN, Polyphen2, SIFT and phastCons, were reported to be effective to determine the deleteriousness of missense variants^9^. We also calculated the mean scores of ClinVar pathogenic missense variants for CADD (mean ± SD = 28.04 ± 5.76), DANN (mean ± SD = 0.99 ± 0.06), fathmm_MKL_coding (mean ± SD = 0.90 ± 0.18), phastCons (mean ± SD = 0.84 ± 0.29), Polyphen2 (mean ± SD = 0.90 ± 0.25), SIFT (mean ± SD = 0.04 ± 0.12) and VEST3 (mean ± SD = 0.78 ± 0.24) (Supplementary Dataset 4). We applied these mean scores as cut-offs with which to differentiate deleterious from non-deleterious variants when evaluating the other missense variants in the list of 2699 genes. If a missense variant receives a score equal to or above (or below for SIFT) the mean values for at least five out of the seven tools, it was retained for downstream filtering. Additional criteria were applied to the filtering (see Methods). Overall, 48,634 Type 3 variants were identified (Supplementary Dataset 3).

We employed an alternative tool, EVE, to assess the deleteriousness of missense variants identified. EVE is a model for the prediction of clinical significance of human variants based on sequences of diverse organisms across evolution, which used fully unsupervised deep learning trained on amino acid sequences of over 140 K species^16^. EVE classifications were available for missense variants in 2208 genes, among which 17,136 variants from 650 genes overlapped with our defined Type 3 variant list. Reassuringly, only 0.54% (92 out of 17,136) of these variants were classified as Benign by EVE, demonstrating the reliability of our method in determining the pathogenicity of missense changes.

#### Type 4 variants – potentially harmful in-frame insertion and deletion mutations (INDELs)

In-frame INDELs are much less common than LoF or missense changes. Following our Type 3 variant analysis strategy, only genes with known ClinVar pathogenic in-frame INDELs were included. If there is no ClinVar pathogenic in-frame INDEL variant for a specific gene, there would be zero Type 4 variant. Consequently, 8887 such variants in 1654 genes remained (Supplementary Dataset 5). Further, variants that are evolutionarily conserved (CADD score > 20^17^), located in functionally critical domains and in close proximity to known pathogenic in-frame INDELs were denoted as Type 4 variants (see Methods). Altogether, 535 Type 4 variants were identified (Supplementary Dataset 3).

### Ethnicity-specific ranking of carrier frequencies in the discovery cohort

**Fig. 3:**
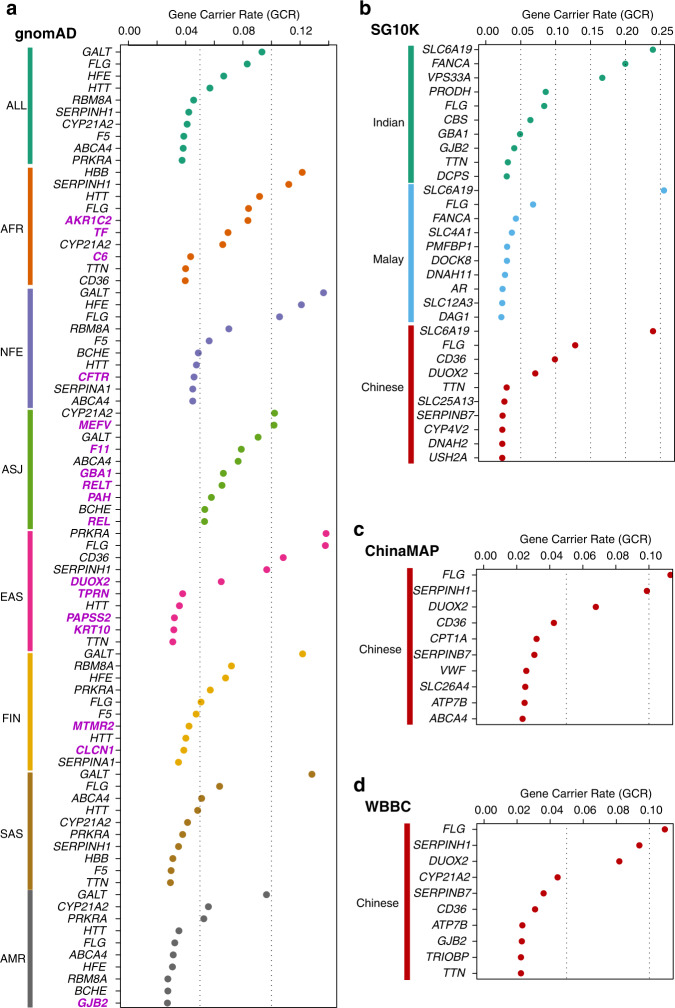
Gene carrier rates (GCRs) for the top ten genes of each ethnicity within each cohort. Genes are listed on the vertical axis, and the GCR values are shown on the horizontal axis. **a** gnomAD cohort containing GCRs from seven subpopulations. ALL all gnomAD samples, AFR African/African American, NFE European (non-Finnish), ASJ Ashkenazi Jewish, EAS East Asian, FIN European (Finnish); SAS South Asian, AMR Latino/Admixed American. Note that ethnicity-specific top gene(s) not in any other ethnicity’s top 10 gene lists were highlighted in bold with purple color. **b** Singapore cohort containing GCRs from three subpopulations. **c** ChinaMAP cohort and **d** WBBC cohort both composed of Chinese population GCRs.

A combined list of Type 1 through Type 4 gnomAD variants were generated for each of the 2699 genes (Fig. 1). Ethnicity-specific variant carrier rate (VCR) was calculated for all filtered variants in each gene, and the ethnicity-specific gene carrier rates (GCR) were subsequently deduced (see Methods for calculation formula). Because seven sets of VCRs were identified for each ethnicity in each gene, this calculation yielded seven sets of ethnicity-specific GCRs (Fig. 1 middle panel, Supplementary Dataset 6). Genes were then sorted based on the descending GCR values for each ethnicity. As a result, genes with the highest GCRs, and therefore those with the highest probabilities of causing recessive diseases in offspring, were at the top of the list for each population (Fig. 1 lower panel). GCRs for the top ten genes in each population were illustrated **(**Fig. 3a).

Since prediction by in silico tools is prone to error, to evaluate the contribution of Type 3 and Type 4 variants to the overall result, we calculated GCR rankings based on Type 1 and Type 2 variants only and compared the values to GCR rankings based on all Type 1 to Type 4 variants in the gnomAD database (Supplementary Fig. 2). Nearly identical rankings were observed (Pearson correlation coefficient *R* = 0.95 to 0.98, Pearson correlation coefficient *R* = 0.88 to 0.93), presumably due to the stringent filtering criteria set for Type 3 and Type 4 changes, which resulted in small amount of such variants being retained and their marginal contribution to the overall result. Therefore, we included all Type 1 to Type 4 variants for the remaining analysis to avoid neglecting missense and inframe INDELs changes.

### Ranking of carrier frequencies in validation cohorts

To verify the above pipeline in ranking carrier frequencies, we further analyzed variants from three independent genome databases with WGS data on large scale East Asian (Chinese) and South Asian (Malay and Indian) populations. These databases included the Singapore 10 K Genome Project (SG10K)^18^, the China Metabolic Analytics Project (ChinaMAP)^19^ and the Westlake BioBank for Chinese (WBBC) pilot project^20^.

**Fig. 4:**
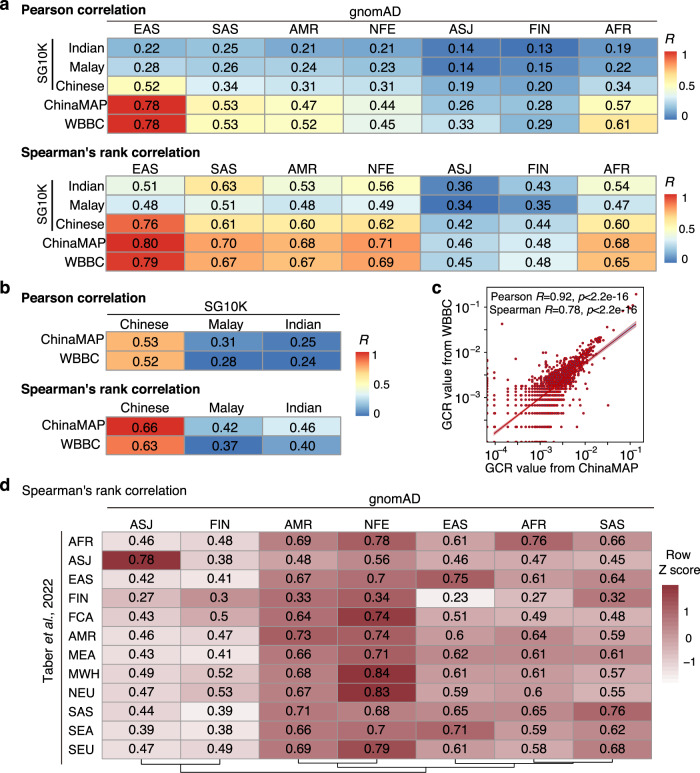
Comparison of carrier frequencies among different cohorts. **a** Comparison between gnomAD populations and SG10K subpopulations, ChinaMAP Chinese or WBBC Chinese. Spearman’s rank correlation coefficient and Pearson correlation coefficient *R* scores are illustrated in each cell and color coded. Red indicates high correlation, while blue indicates low correlation. **b** Comparison between SG10K subpopulations and ChinaMAP Chinese or WBBC Chinese. **c** Comparison between ChinaMAP Chinese and WBBC Chinese. See also Supplementary Fig. 4 for comparison scatter plots and corresponding statistical *P* values. **d** Spearman’s correlation coefficients generated between our calculated carrier frequencies of gnomAD populations and actual ethnicity specific carrier frequencies recorded from NGS based ECS (Taber *et al*. 2022 study, ref. ^6^). AFR African or African-American, ASJ Ashkenazi Jewish, MWH Mixed or Other White, FCA French Canadian or Cajun, EAS East Asian, FIN Finnish, AMR Hispanic (corresponding to the Latino/Admixed American population in gnomAD), MEA Middle Eastern, NEU Northern European, SAS South Asian, SEA Southeast Asian, SEU Southern European. The color stands for row-wise Z score scaled Spearman’s correlation coefficient. See also Supplementary Fig. 5 for Pearson correlation coefficients.

WGS data of 4810 SG10 K samples from three Asian subpopulations (2780 Chinese, 903 Malays and 1127 Indians) were obtained. After removing outlier samples from each subpopulation (see Methods), WGS data from 2613 Chinese, 721 Malay and 1001 Indian were retained for downstream analysis (Supplementary Fig. 3). Type 1 to Type 4 variants were identified based on the criteria described above (Supplementary Dataset 3), and subpopulation-specific GCRs were calculated for the 2699 genes and ranked subsequently (Supplementary Dataset 6, Fig. 3b). We compared the rankings of the 2699 genes in SG10 K subpopulations with those in the gnomAD database. As expected, among the seven gnomAD ethnicities, the ranking in SG10K Chinese correlated with gnomAD East Asian the most (Fig. 4a, Supplementary Fig. 4, Spearman rank correlation coefficient *R* = 0.76, Pearson correlation coefficient *R* = 0.52), while the ranking in SG10K Indian correlated with gnomAD South Asian the most (Fig. 4a, Supplementary Fig. 4, Spearman *R* = 0.63, Pearson *R* = 0.25). Ranking in SG10K Malay only showed slightly higher correlation with gnomAD South Asian than other ethnicities, presumably due to its small sample size (721 individuals only) and the distinct genetic background between Singapore Malay and self-reported South Asian resided in the U.S.

We also calculated the GCR ranking of the 2699 genes in the ChinaMAP cohort (Supplementary Dataset 6, Fig. 3c). The ChinaMAP database aggregated WGS data from 10,588 Chinese individuals, majority of which (9043) were Han Chinese. Again, among the seven gnomAD ethnicities, the ranking in ChinaMAP correlated with gnomAD East Asian the most (Fig. 4a, Spearman *R* = 0.80, Pearson *R* = 0.78). Similarly, GCR ranking in the WBBC cohort containing WGS data from 4480 Chinese individuals (Supplementary Dataset 6, Fig. 3d) also showed high correlation with gnomAD East Asian (Fig. 4a, Spearman *R* = 0.79, Pearson *R* = 0.78). In addition, ChinaMAP ranking and WBBC ranking resembled that from the SG10K Chinese, but not that from the SG10K Indian or Malay (Fig. 4b). Notably, when comparing between ChinaMAP and the WBBC cohorts, both of which contained Chinese population resided in China and thus the most similar genetic background among all the databases, their GCR rankings demonstrated exceedingly high correlation (Fig. 4c, Pearson *R* = 0.92, Spearman *R* = 0.78), confirming the robustness of our analysis pipeline.

### Comparison with carrier frequencies from previous studies

We further compared our rankings in the gnomAD cohort to actually observed carrier frequencies from a retrospective NGS based ECS study^6^. This ECS study evaluated carrier frequencies of 176 conditions in >460,000 individuals across 11 self-defined ethnicities. Again as expected, comparison of populations with the same ethnic background between the two cohorts demonstrated high correlation, *e.g*., gnomAD ASJ versus ECS ASJ (Spearman *R* = 0.78, Pearson *R* = 0.81), gnomAD EAS versus ECS EAS (Spearman *R* = 0.75, Pearson *R* = 0.66), gnomAD NFE versus ECS MWH (mixed or other white), NEU (Northern European) and SEU (Southern European) (Spearman *R* = 0.84, 0.83 and 0.79, respectively) (Fig. 4d, Supplementary Fig. 5). These results further validated the reliability of our analysis pipeline.

We also compared our findings to previously published estimation based on genome-wide sequencing data of unaffected individuals (Supplementary Fig. 6). Majority of previous studies on estimated carrier frequencies focused on a single gene or one disease spectrum. We were able to find three studies consisting of analysis on hundreds of recessive genes using gnomAD or gnomAD plus in-house databases, including one analysis on 185 genes associated with AR retinal diseases^10^, one on 249 genes with AR mitochondrial disorders^11^ and one on 415 genes with severe recessive conditions^9^. Except for the relatively low correlation in the Ashkenazi Jewish (ASJ) population between our ranking and the 249 mitochondrial disorder genes study (Spearman *R* = 0.38; no ASJ data was available for the 185 retinal disease genes study), rankings in other ethnicities demonstrate moderate to high correlations, particularly for the NFE population (Spearman *R* = 0.76 when compared with the 185 retinal disease genes study, 0.75 with the 249 mitochondrial genes study and 0.72 with the 415 severe condition genes study).

Additionally, we compared GCRs of two representative genes, *CFTR* and *ABCA4*, to previously studies (Supplementary Dataset 7)^6,7,9,10^. We observed similar carrier frequency values for *CFTR* compared to several published results; while for *ABCA4*, our GCRs are comparable yet slightly higher. We also calculated the predicted genetic prevalence at the gene level (pGPg) based on GCRs from various cohorts (see Methods for calculation equation). Disease prevalence for 405 genes associated with AR monogenic diseases were estimated (Supplementary Dataset 8).

## Discussion

In this study, we established a robust pipeline for estimating and ranking carrier frequencies of all known recessive genes based on genome-wide sequencing data. To overcome the major obstacle on obtaining accurate carrier frequencies, which is the correct annotation of variants with respect to their pathogenicity^21^, we generated comprehensive selection criteria for various kinds of potentially disease-causing variants, including Type 2 LoF, Type 3 missense and Type 4 in-frame INDEL changes. Our filtering criteria were verified to be reliable, which was illustrated by resembling results based on different datasets (Fig. 4a–c), as well as comparable results with previous studies at both single gene level (Supplementary Dataset 7) and overall rankings among different cohorts (Fig. 4d, Supplementary Fig. 6).

Although the general trends of our ranked gene lists for the Asian populations were similar between the discovery and validation cohorts, we noticed the differences in the ranking of some genes (Supplementary Dataset 6, Supplementary Dataset 7). These differences may be attributable to the nature of the individuals recruited in discovery gnomAD cohort, who are US residents of self-reported East or South Asian ethnicity. Therefore, these self-reported ethnicities may not accurately reflect their actual genetic backgrounds, which likely diverged from those of the local residents in the validation cohorts. However, when comparing the rankings between the two Chinese cohorts, ChinaMAP and WBBC, they demonstrated exceedingly high correlation (Fig. 4c, Pearson *R* = 0.92, Spearman *R* = 0.78), supporting the validity of our variant selection criteria and the overall analysis pipeline.

Carrier frequencies of recessive diseases are well-known to vary markedly among different populations. Historically, carrier frequencies of a few common defects were determined through genetic testing of known pathogenic variants^8^. The widespread application of the NGS technique allowed the acquisition of carrier frequencies of much larger number of genes. This was accomplished via two approaches: estimation based on large scale genome-wide sequencing data and actual observation from ECS results. The former estimation approach mostly focused on one gene^22–24^ or one disease spectrum^25,26^, while the later approach was limited to the genes included in the ECS panel^5,6^. Our current study installed a comprehensive analysis on all known recessive genes. Further, our analysis pipeline is readily adaptable to prospective novel recessive genes. As a matter of fact, it was estimated that the overall number of AR genes with recognizable phenotypes lies between 9000 and 10,100, suggesting that the currently known AR genes represent only ~20% of the total^27^.

NBS program enabled early diagnosis and initiation of effective treatment to ameliorate the adverse outcome of many disorders, of which the vast majority are recessive IEM^4^. Regional-specific carrier frequencies of the local population should be an integral determinant for effective selection of a territory NBS panel. For example, consider a recent pilot study of NBS for 24 IEM diseases in Hong Kong^28^, a region with an ethnic composition of 86.5% of the newborns being Chinese (East Asian) and the remaining infants mostly being Southeast or South Asian (Filipino, Indian, Nepalese or Pakistani)^29^. This 18-month retrospective study^28^ recorded nine positive IEM patients with six diseases (Supplementary Dataset 9). Our analysis demonstrated that carrier frequencies of these six diseases were indeed high in the East Asian and South Asian populations (Supplementary Dataset 9). Specifically, diseases such as citrullinemia type II and carnitine uptake deficiency, which were confirmed in more than one Chinese patients, ranked as high as 14 and 12 among carrier frequencies of all 2699 recessive genes in the WBBC Chinese cohort. Similarly, these two genes ranked 21 and 20 in the ChinaMAP Chinese cohort. Nevertheless, some of the remaining 18 diseases without identified positive cases had rather low carrier frequency rates in both East and South Asian populations (Supplementary Dataset 9), indicating a more effective selection of NBS panel for the region is warranted.

The design of NBS and ECS panels is a delicate balance of comprehensiveness versus cost-effectiveness. For NBS, only treatable diseases with relatively high prevalence should be included. Whereas for ECS, with the reduction of NGS cost, increasing numbers of genes are being added to different panels, with some suggested whole exome or even whole genome sequencing for preconception carrier screening^30,31^. However, ECS cannot be described as “the more, the merrier”. Larger panels result in a lower sequencing depth for individual genes and thus lead to missed variant calling^32^. Moreover, these panels place unnecessary burdens on variant interpretation and genetic counselling^33,34^, which are the two most time and financially consuming processes. Therefore, genes and diseases included in NBS and ECS panels should be precisely determined and tailored for the situation in each region or territory. Recent guidelines^6,35^ for a pan-ethnic, universal ECS panel may be suitable for mixed races countries like the U.S. with high possibility of interracial couples to produce mixed-race children. For countries and regions with a single majority ethnicity group, a more focused panel would be more economically efficient yet sufficient.

Notably, the NGS-based datasets utilized in our analysis generally only detect short sequence changes such as SNVs (single nucleotide variants). High-prevalence yet technically challenging variant types, *e.g*. CNVs (copy-number variations) in spinal muscular atrophy, repeat expansions in the Fragile X syndrome and the highly repetitive and purine-rich ORF15 region in *RPGR*, were not included^35,36^. We also note that in addition to carrier frequencies, other criteria for NBS and ECS panel selection should be considered. Specifically, for NBS, supplementary criteria should include the availability of reliable laboratory screening and diagnostic testing methods, seriousness of the disease, availability of early treatment, and favorability of the post-intervention outcome^4^. For ECS, additional considerations for scrutiny should include the severity of the disease, possibility of preimplantation genetic diagnosis, availability of prenatal diagnostic testing, and sufficient knowledge of genotype–phenotype correlations^37^.

In summary, we established a robust pipeline for estimating and ranking carrier frequencies, which is readily adaptable to new genome-wide sequencing data and to prospective novel recessive genes. Since carrier frequencies in a given population would be one of the most critically considered aspects for NBS and ECS design, our data-driven analysis provides a scientific basis and establish guidelines for such practices.

## Methods

### Extraction of AR and XL genes

Known disease-causing recessive genes included for calculation in this study were obtained from the OMIM database (https://www.omim.org/, last enquired on September 27, 2022). The genemap2.txt file was processed and genes were retained if simultaneously fulfilling the following criteria in column “Phenotypes”:

1. Contain “(3)” as the phenotype mapping key, which is defined as “the molecular basis of the disorder is known” by OMIM;
2. Contain “Autosomal recessive” or “X-linked”; genes were further removed if only “X-linked dominant” was annotated;
3. Contain at least one sub-phenotype that does not begin with “?”, “{” or “[”.

Eventually, 2699 recessive genes were retained, including 2525 on autosomal chromosomes and 174 on chromosome X (Supplementary Dataset 2).

### Filtering and annotation of gnomAD variants

Compressed Variant Call Format (VCF) files from gnomAD exome v211 (GRCh38 liftover) containing 125,748 exomes and gnomAD genome v3.1.1 containing 76,156 genomes were obtained from the gnomAD database (http://gnomad.broadinstitute, downloaded on July 27, 2021; note that the 15,708 gnomeAD v2 genomes were not included in this study to avoid overlapping individuals being sequenced in the v3 genome cohort). Only high-quality variants simultaneously fulfilling the criteria below were retained for downstream analysis:

1. Labeled as “PASS” in the VCF files;
2. Covered in more than 50% individuals in the corresponding dataset.

Subsequently, the allele count (AC), allele number (AN) and number of individuals with homozygous alternative variant (when available) of each high-quality variant from gnomAD exome v2.1.1 and genome v3.1.1 were summed up within each ethnicity group. Allele frequencies were calculated based on the merged AC and AN values. Variants were annotated using ANNOVAR^38^, which contained information from the ClinVar database (version 3.5a on July 24, 2021). Gene annotation file was downloaded from the UCSC Genome Browser (https://genome.ucsc.edu/, “NCBI RefSeq” track). Coordinates of the start/stop codon of each gene and the length of the coding DNA sequence were deduced based on “NM_” protein coding transcripts.

Notably, 19 variants (Supplementary Dataset 10) were excluded from the analyses following previous studies^5,9^ due to high AF ( ≥ 0.005) in at least one gnomAD population, of known low penetrance or poor sequencing quality.

### Selection of Type 1 variants

Among high-quality variants from gnomAD, 27,515 (0.02%) were reported as pathogenic/likely pathogenic (P/LP), 162,462 (0.13%) as benign/likely benign (B/LB), 155,743 (0.12%) as VUS, and 34,210 (0.03%) as others (*e.g*., conflicting data from submitters, associations, risk factors, protective, drug response). Note that pathogenic changes documented in ClinVar can be of any variant type, including frameshift, missense, nonsense, splice change, non-coding RNA, structural variants, *etc*. Among the P/LP ClinVar variants (curation as on Jul 24, 2021), we manually reviewed those with AF ≥ 0.005 and removed 10 variants with updated ClinVar category being B or conflicting (last queried on Oct 14, 2022) to avoid inflating of our GCR values (Supplementary Dataset 10).

We noticed that some variants with conflicting ClinVar curations were due to one or a few benign entries, while the overwhelming majority of the entries were actually pathogenic. For example, the well-established pathogenic change p.Glu7Val in *HBB* causing sickle cell anemia has 32 P and 4 B/LB ClinVar entries, while the p.Cys282Tyr in *HFE* causing hemochromatosis has 26 P and 1 VUS ClinVar entries (last queried on Oct 14, 2022). Therefore, we kept ClinVar conflicting variants with no less than 10 pathogenic entries. 61 such variants were retained. As expected, very few B/LB or VUS curations were documented for these variants. Among them, we manually removed five variants with AF ≥ 0.005 AND were known to have low penetrance, being a risk allele only, and/or not disease causing in homozygous status (Supplementary Dataset 10). The remaining 56 variants were annotated as Type 1 changes.

### Additional selection criteria of Type 3 variants

As described in the Results section, we applied mean scores from seven tools as cut-offs to differentiate deleterious from non-deleterious missense variants. Of the seven tools, five tools (CADD, DANN, Polyphen2, SIFT and phastCons) were required to be always included since they were reported to be effective^9^. To further select potentially deleterious changes, we applied more stringent criteria to the filtering of missense variants. Specifically, only variants in genes with known pathogenic missense changes in ClinVar were included. If there is no ClinVar pathogenic missense variant for a specific gene, there would be zero Type 3 variant. 2030 out of the 2699 genes met this criteria (Supplementary Dataset 5). For the 2030 genes that fulfil the above requirement, we calculated the gene-specific missense mean scores using the seven prediction tools, and only missense variants with scores that met at least five gene-specific cut-offs were kept as Type 3 variants. For genes with gene-specific cut-offs lower than the mean scores of total gnomAD ClinVar P/LP missense variants, the latter were applied for filtering. In other words, we always followed the more stringent cut-off scores. Overall, 48,634 Type 3 variants were identified (Supplementary Dataset 3).

### EVE classification of Type 3 variants

We set 25% of all possible variants in EVE prediction as uncertain, which would result in an accuracy of approximately 90% for pathogenic and benign classifications^16^. Of the 2208 genes with available EVE prediction, 650 genes overlapped with our recessive gene list. Among the 17,136 gnomAD Type 3 variants in the 650 genes, EVE classified 92 as Benign (0.54%).

### Functionally critical domains for defining Type 4 variants

Protein functional domains were determined according to the Pfam database^39^ (downloaded from the UCSC Genome Browser on July 9, 2021, “Pfam in GENCODE” track). In-frame INDELs located in the same domain with known ClinVar P/LP in-frame INDELs were retained.

### Calculation of VCR and GCR

A combined list of Type 1 through Type 4 variants were generated for each of the 2699 genes. Ethnicity-specific allele count (AC) and total number of alleles analyzed (AN) for each variant, as well as the numbers of individuals who are homozygous for the variant (Hom) were extracted for calculation. The ethnicity-specific variant carrier rate (VCR) was calculated using the following equation:

$$VCR = \frac{{AC - 2 \times Hom}}{{0.5 \times AN}}$$

As a result, 2699 lists of VCRs for the selected deleterious variants were calculated, and each list comprised the ethnicity-specific VCRs of each variant.

The ethnicity-specific gene carrier rates (GCR) for each of the 2699 genes were calculated using the following equation:

$$GCR = 1 - \mathop {\prod }\limits_{i = 1}^n \left( {1 - VCRi} \right)$$

Here, VCRi represents the variant carrier rate for variant i, and n represents the number of variants selected in this particular gene.

The predicted genetic prevalence at the gene level (pGPg) was calculated using the following equation:

$$pGPg = \frac{{\mathop {\sum}\nolimits_{k = 1}^n {\left( {VCR} \right)ik\left( {VCR} \right)ik} }}{4}$$

### SG10K cohort

The SG10K Project sequenced 4810 samples from three Asian subpopulations, including 2780 Chinese, 903 Malays and 1127 Indians^18^. VCF files were downloaded from the European Genome phenome Archive (EGA) under accession number EGAS00001003875 with permission from the authors. The VCF files contained genotype information of each individual. To remove outlier samples from each subpopulation within the cohort, smartpca from EIGENSOFT v 6.1.4^40^ was applied to perform principal component analysis (PCA) on individual genotypes from SG10K and The 1000 genome project with East Asian ancestry and South Asian ancestry^41^. Outlier samples that were outsider of the center of certain populations were removed. Eventually, 2613 Chinese, 721 Malay and 1001 Indian individuals were retained for downstream analysis (Supplementary Fig. 3).

### ChinaMAP cohort

The ChinaMAP cohort contained deep WGS data (40.80×) from 10,588 Chinese participants, the majority (9043, 85.41%) of which were HAN samples^19^. VCF files were downloaded from the ChinaMAP website (http://www.mbiobank.com/download/) on Aug 4, 2021. VCF files contained the AC and AN information for each variant, while only variants located on autosomal chromosomes were listed. All variants were processed similarly to gnomAD data as described above. The information on Hom for the filtered Type 1 to Type 4 variants was later given by the authors of the ChinaMAP project.

### WBBC pilot cohort

The WBBC pilot project contained WGS data (13.9×) from 4480 Chinese individuals^20^. VCF files were downloaded from the WBBC website (https://wbbc.westlake.edu.cn/downloads.html) on May 27, 2022. VCF files contained the AC, AN and Hom information for each variant. All variants were processed similarly to gnomAD data as described above.

### Correlation analysis of GCR

Spearman’s rank correlation coefficient with *P* value and Pearson correlation coefficient with *P* value were calculated using the R package ggpubr 0.4.0 (https://CRAN.R-project.org/package=ggpubr). For correlation analysis between two datasets, only genes with GCR > 0 in at least one dataset were included. Statistics were performed using R 3.6.0 and ggpubr 0.4.0.

### Reporting summary

Further information on research design is available in the Nature Research Reporting Summary linked to this article.

## Supplementary information

Supplementary Figures 1 to 6

Dataset S1

Dataset S2

Dataset S3

Dataset S4

Dataset S5

Dataset S6

Dataset S7

Dataset S8

Dataset S9

Dataset S10

Dataset S11

Reporting Summary Checklist

## Data Availability

Publicly available VCF variant files of human genome projects were downloaded from gnomAD (https://gnomad.broadinstitute.org/downloads), ChinaMAP (http://www.mbiobank.com/), and WBBC (https://wbbc.westlake.edu.cn/downloads.html), respectively (Supplementary Dataset 11). VCF files of SG10K were downloaded from The European Genome-phenome Archive (EGA) under accession number EGAS00001003875 after permission approval by the Data Access Committee for SG10K_Pilot Dataset. Carrier frequencies of the ECS study were collected from the published paper^6^. Publicly available GCR value of 185 genes associated with AR retinal diseases^10^, 249 genes with AR mitochondrial disorders^11^ and 415 genes with severe recessive conditions^9^ were downloaded from corresponding published papers.

## Code availability

Analyses and plotting were performed using R 3.6.0, ggpubr 0.4.0 and pheatmap 1.0.12. Code for drawing panels in Fig. 3 was publicly available at https://github.com/mhguo1/ECS/. Processed data and codes for all analyses described in this study will be available upon request.
