# Supplementary material for: A robust pipeline for ranking carrier frequencies of autosomal recessive and X-linked Mendelian disorders
Source: NPJ Genom Med. 2022 Dec 19;7:72. doi: 10.1038/s41525-022-00344-7 (PMC9763236; doi:10.1038/s41525-022-00344-7)
Supplement: Supplementary file 1 — Supplementary Figures 1 to 6 [file 41525_2022_344_MOESM1_ESM.pdf]

Supplementary figures and legends

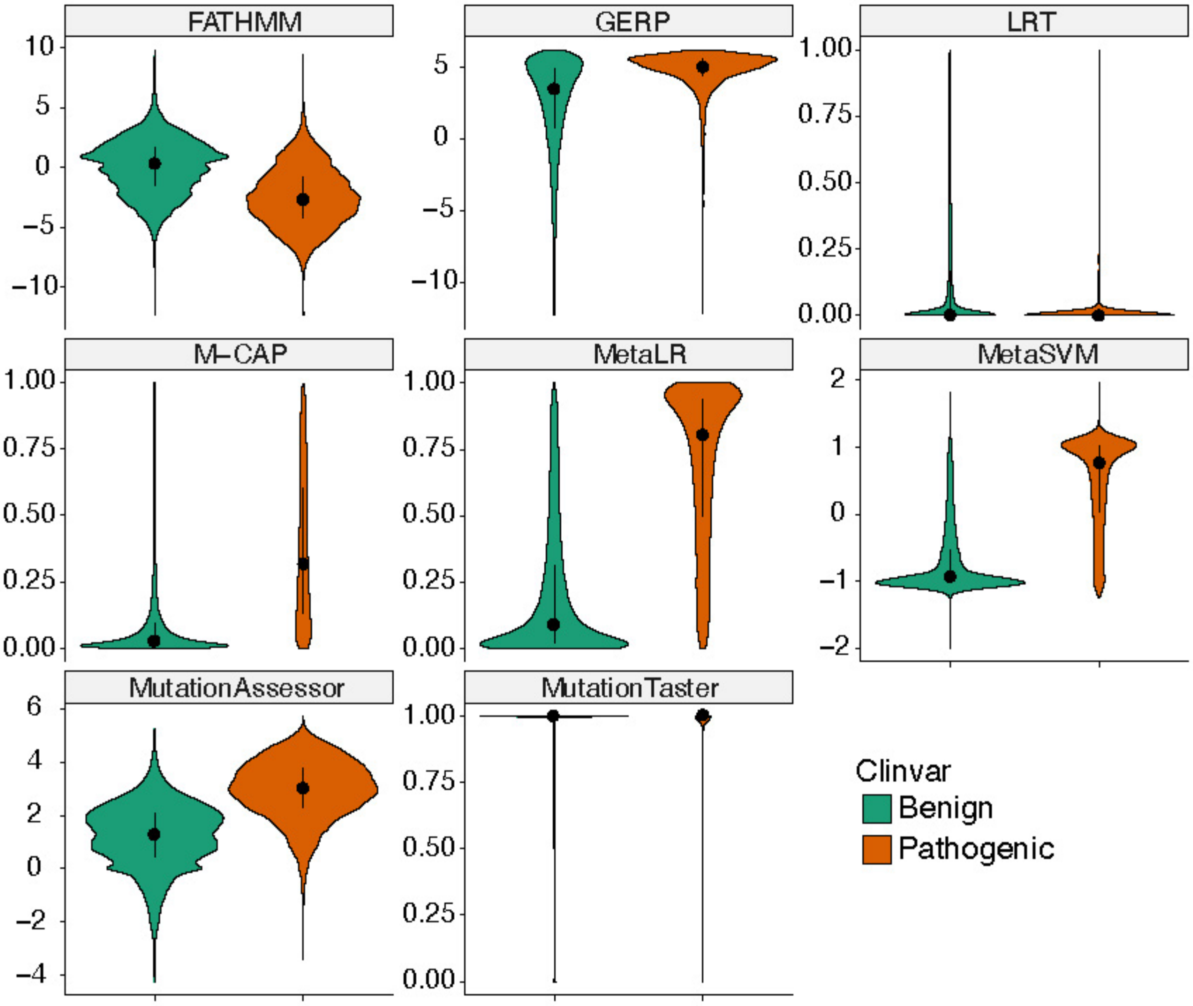

Supplementary Figure 1. Violin plots comparing scores of the remaining eight variant analysis tools. Calculated mean scores with standard deviations are listed in **Supplementary Dataset 4**. See also **Fig. 2**.

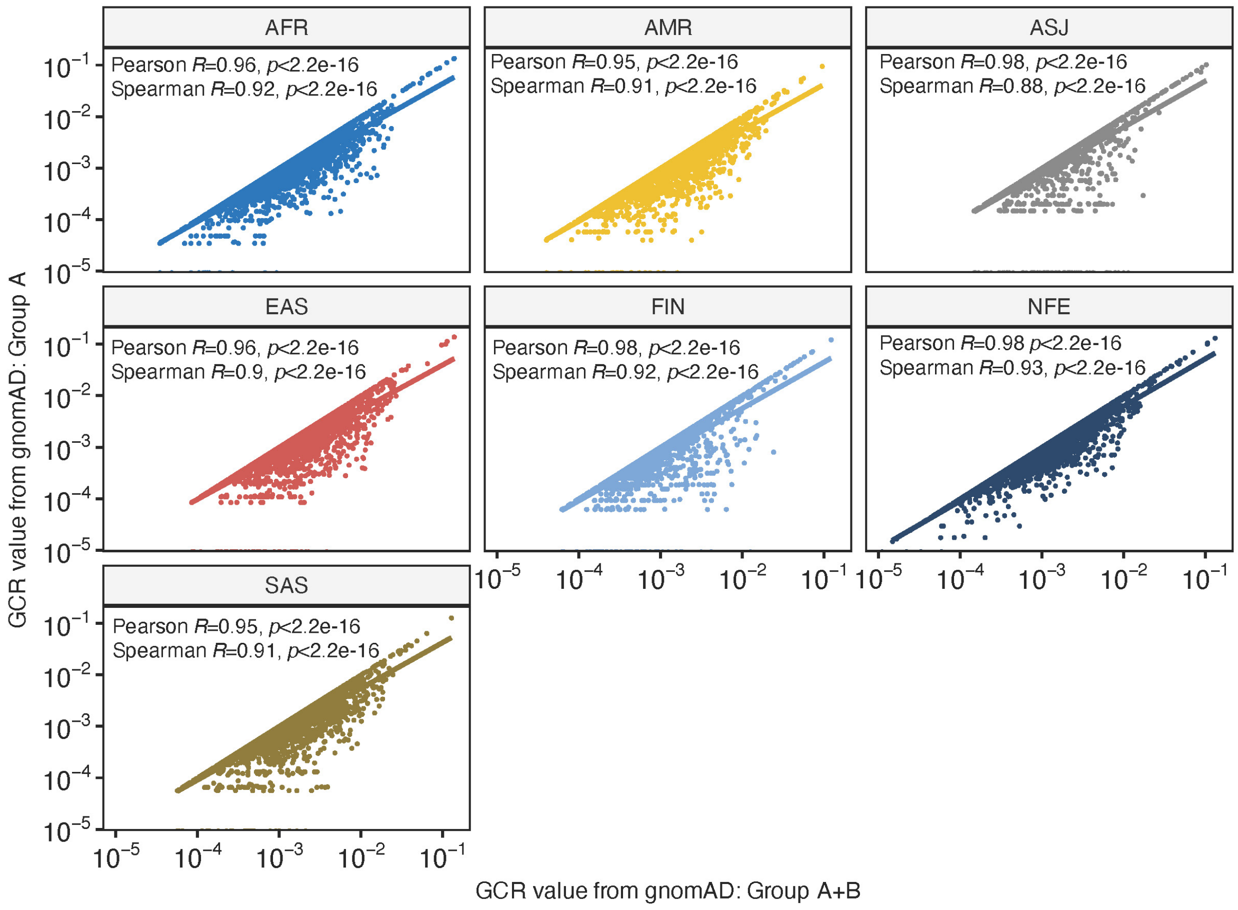

Supplementary Figure 2. Comparison scatter plots and corresponding statistical *P* values of GCR rankings based on Type 1 and Type 2 or Type 1 to Type 4 variants in gnomAD database. Group A refers to Type 1 and Type 2 variants, while Group B refers to Type 3 and Type 4 variants.

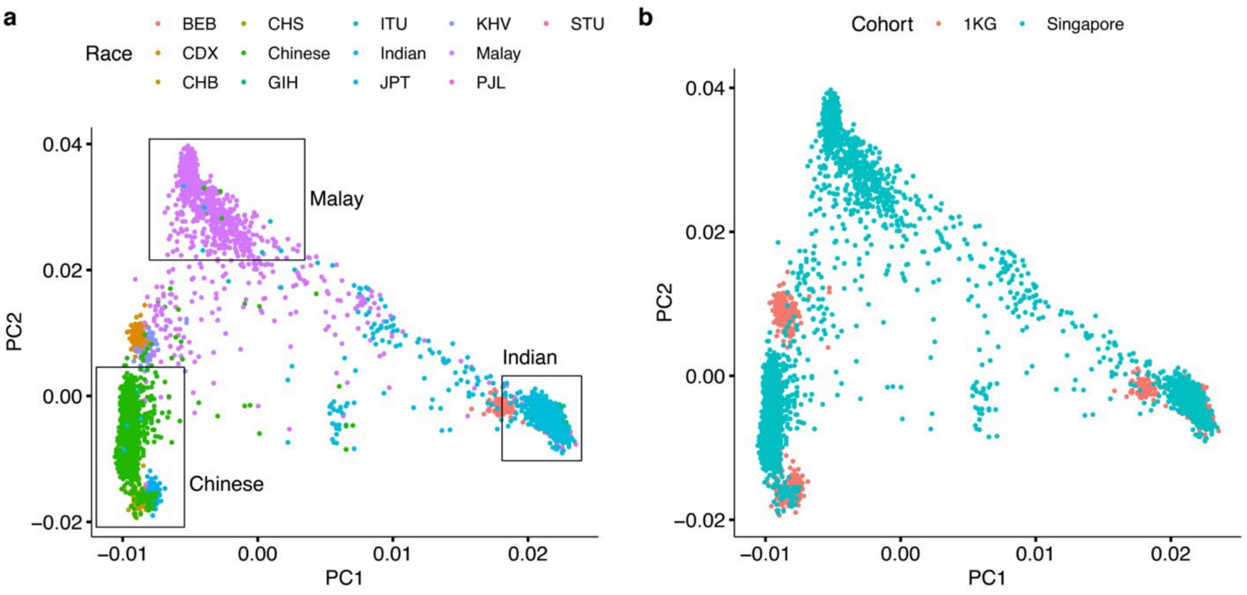

Supplementary Figure 3. PCA analysis removing outliers in each subpopulation in the Singapore (SG10K) database. 1KG: the 1000 genome project; BEB: 1KG subpopulation Bengali in Bangladesh; CHS: 1KG subpopulation Southern Han Chinese, China; ITU: 1KG subpopulation Indian Telugu in the UK; KHV: 1KG subpopulation Kinh in Ho Chi Minh City, Vietnam; STU: 1KG subpopulation Sri Lankan Tamil in the UK; CDX: 1KG subpopulation Chinese Dai in Xishuangbanna, China; CHB: 1KG subpopulation Han Chinese in Bejing, China; GIH: 1KG subpopulation Gujarati Indian in Houston, Texas; JPT: 1KG subpopulation Japanese in Tokyo, Japan; PJL: 1KG subpopulation Punjabi in Lahore, Pakistan.

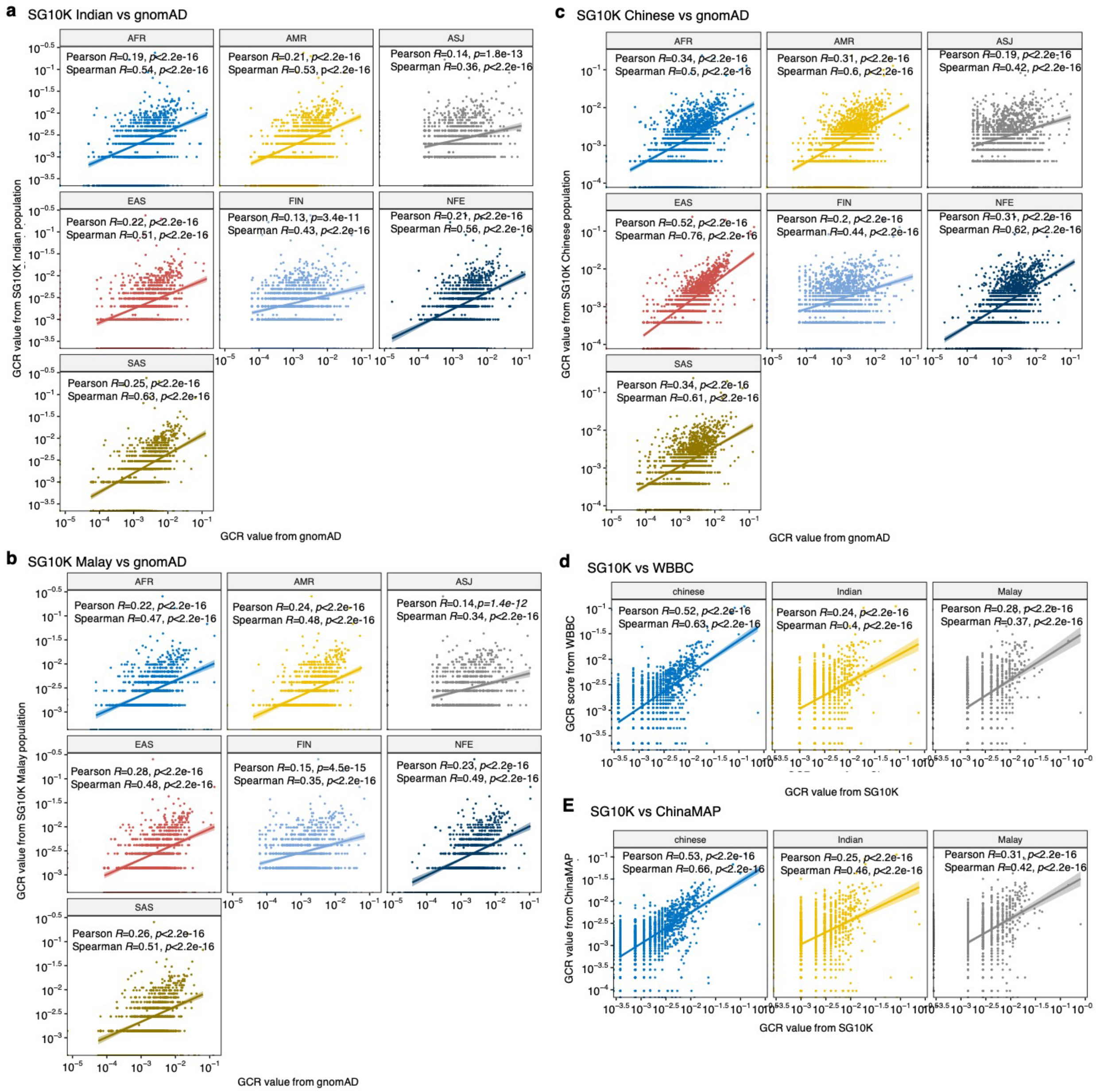

Supplementary Figure 4. Comparison scatter plots and corresponding statistical *P* values on correlation of carrier frequencies among different cohorts. a Comparison between gnomAD populations and SG10K Indian. **b** Comparison between gnomAD populations and SG10K Malay. **c** Comparison between gnomAD populations and SG10K Chinese. **d** Comparison between SG10K subpopulations and WBBC. **e** Comparison between SG10K subpopulations and ChinaMAP. See also **Fig. 4**.

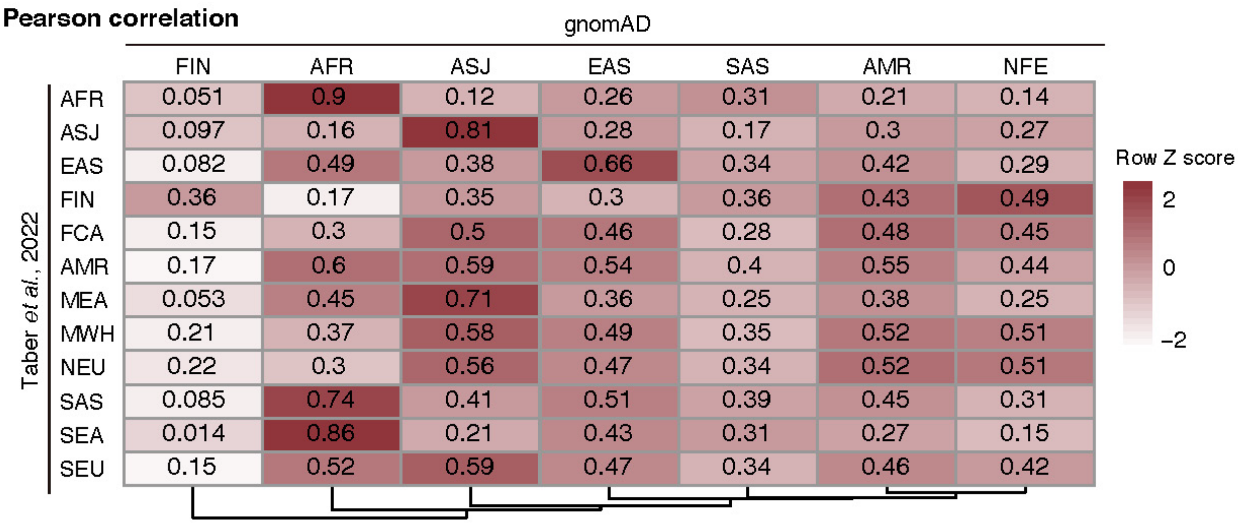

Supplementary Figure 5. Pearson correlation coefficients generated between the calculated carrier frequencies of gnomAD populations and actual ethnicity specific carrier frequencies recorded from NGS based ECS. See also **Fig. 4**.

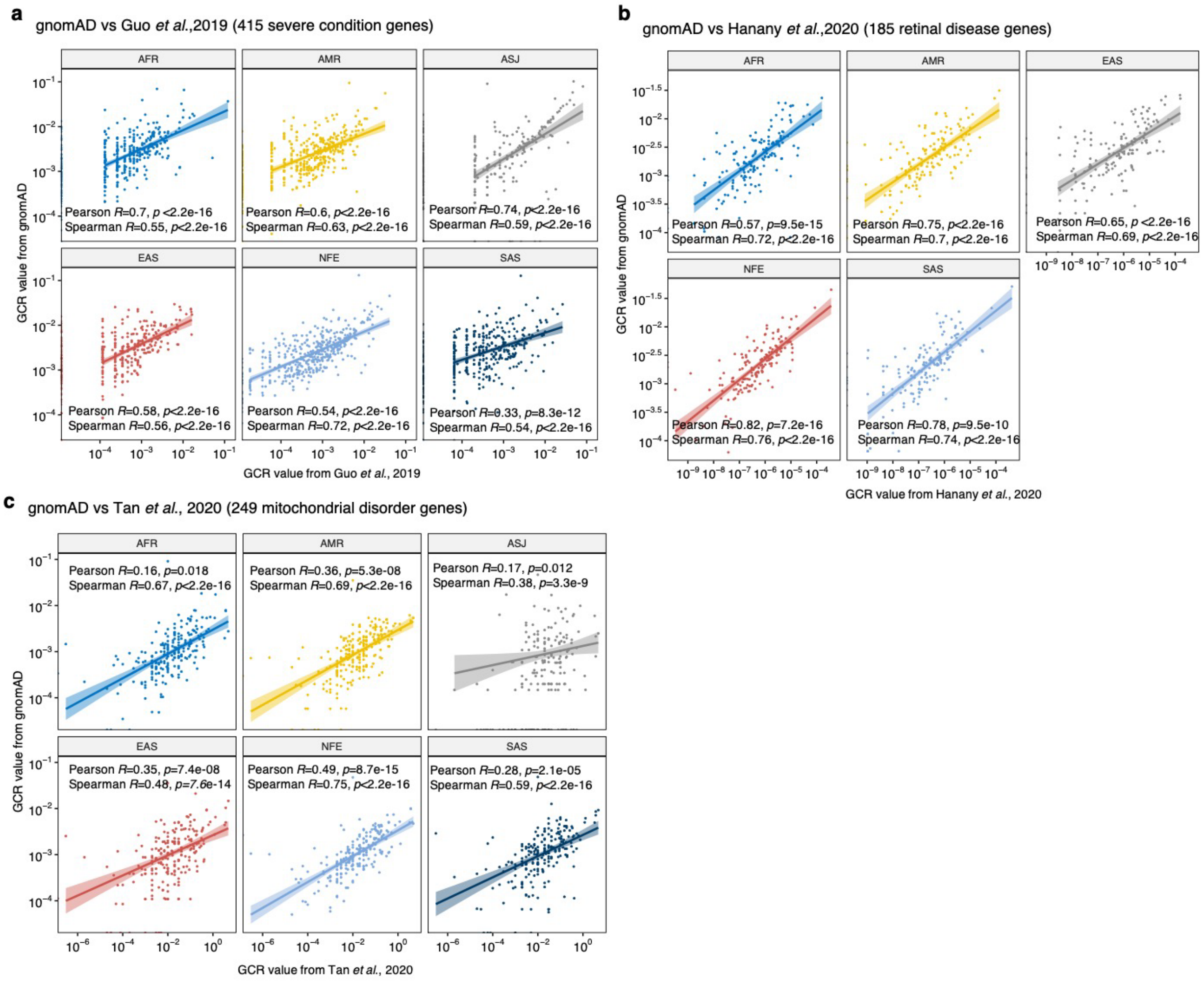

Supplementary Figure 6. Comparison scatter plots and corresponding statistical *P* values on ranking of the calculated gnomAD carrier frequencies with previously published estimations. a Comparison analysis on 415 genes with severe recessive conditions. **b** Comparison analysis on 185 genes associated with AR retinal diseases. **c** Comparison analysis on 249 genes with AR mitochondrial disorders.
