# Supplementary material for: A robust pipeline for ranking carrier frequencies of autosomal recessive and X-linked Mendelian disorders
Source: NPJ Genom Med. 2022 Dec 19;7:72. doi: 10.1038/s41525-022-00344-7 (PMC9763236; doi:10.1038/s41525-022-00344-7)
Supplement: Supplementary file 13 — Reporting Summary Checklist [file 41525_2022_344_MOESM13_ESM.pdf]

nature portfolio

Corresponding author(s): Shen Gu

Last updated by author(s): Oct 18, 2022

Reporting Summary

Nature Portfolio wishes to improve the reproducibility of the work that we publish. This form provides structure for consistency and transparency in reporting. For further information on Nature Portfolio policies, see our Editorial Policies and the Editorial Policy Checklist.

Statistics

For all statistical analyses, confirm that the following items are present in the figure legend, table legend, main text, or Methods section.

n/a	Confirmed	
☐	☒	The exact sample size (n) for each experimental group/condition, given as a discrete number and unit of measurement
☐	☒	A statement on whether measurements were taken from distinct samples or whether the same sample was measured repeatedly
☐	☒	The statistical test(s) used AND whether they are one- or two-sided<br>*Only common tests should be described solely by name; describe more complex techniques in the Methods section.*
☒	☐	A description of all covariates tested
☒	☐	A description of any assumptions or corrections, such as tests of normality and adjustment for multiple comparisons
☐	☒	A full description of the statistical parameters including central tendency (e.g. means) or other basic estimates (e.g. regression coefficient) AND variation (e.g. standard deviation) or associated estimates of uncertainty (e.g. confidence intervals)
☒	☐	For null hypothesis testing, the test statistic (e.g. F, t, r) with confidence intervals, effect sizes, degrees of freedom and P value noted<br>*Give P values as exact values whenever suitable.*
☒	☐	For Bayesian analysis, information on the choice of priors and Markov chain Monte Carlo settings
☒	☐	For hierarchical and complex designs, identification of the appropriate level for tests and full reporting of outcomes
☐	☒	Estimates of effect sizes (e.g. Cohen's d, Pearson's r), indicating how they were calculated

Our web collection on statistics for biologists contains articles on many of the points above.

Software and code

Policy information about availability of computer code

Data collection	Analyses and plotting were performed using R 3.6.0, ggpubr 0.4.0 and pheatmap 1.0.12. Code for drawing panels in Fig. 3 was publicly available at https://github.com/mhguo1/ECS/.
Data analysis	Processed data and codes for all analyses described in this study will be available upon request.

For manuscripts utilizing custom algorithms or software that are central to the research but not yet described in published literature, software must be made available to editors and reviewers. We strongly encourage code deposition in a community repository (e.g. GitHub). See the Nature Portfolio guidelines for submitting code & software for further information.

Data

Policy information about availability of data

All manuscripts must include a data availability statement. This statement should provide the following information, where applicable:

- Accession codes, unique identifiers, or web links for publicly available datasets
- A description of any restrictions on data availability
- For clinical datasets or third party data, please ensure that the statement adheres to our policy

Publicly available VCF variant files of human genome projects were downloaded from gnomAD (https://gnomad.broadinstitute.org/downloads), ChinaMAP (http://www.mbiobank.com/), and WBBC (https://wbbc.westlake.edu.cn/downloads.html), respectively (Table S11). VCF files of SG10K were downloaded from The European Genome-phenome Archive (EGA) under accession number EGAS00001003875 after permission approval by the Data Access Committee for SG10K_Pilot

Dataset. Carrier frequencies of the ECS study were collected from the published paper. Publicly available GCR value of 185 genes associated with AR retinal diseases, 249 genes with AR mitochondrial disorders and 415 genes with severe recessive conditions were downloaded from corresponding published papers.

Human research participants

Policy information about studies involving human research participants and Sex and Gender in Research.

Reporting on sex and gender	*Use the terms sex (biological attribute) and gender (shaped by social and cultural circumstances) carefully in order to avoid confusing both terms. Indicate if findings apply to only one sex or gender; describe whether sex and gender were considered in study design whether sex and/or gender was determined based on self-reporting or assigned and methods used. Provide in the source data disaggregated sex and gender data where this information has been collected, and consent has been obtained for sharing of individual-level data; provide overall numbers in this Reporting Summary. Please state if this information has not been collected. Report sex- and gender-based analyses where performed, justify reasons for lack of sex- and gender-based analysis.*
Population characteristics	*Describe the covariate-relevant population characteristics of the human research participants (e.g. age, genotypic information, past and current diagnosis and treatment categories). If you filled out the behavioural & social sciences study design questions and have nothing to add here, write "See above."*
Recruitment	*Describe how participants were recruited. Outline any potential self-selection bias or other biases that may be present and how these are likely to impact results.*
Ethics oversight	*Identify the organization(s) that approved the study protocol.*

Note that full information on the approval of the study protocol must also be provided in the manuscript.

Field-specific reporting

Please select the one below that is the best fit for your research. If you are not sure, read the appropriate sections before making your selection.

☒ Life sciences ☐ Behavioural & social sciences ☐ Ecological, evolutionary & environmental sciences

For a reference copy of the document with all sections, see nature.com/documents/nr-reporting-summary-flat.pdf

Life sciences study design

All studies must disclose on these points even when the disclosure is negative.

Sample size	The discovery gnomAD cohort contained sequencing data on 76,156 genomes and 125,748 exomes from individuals with seven ethnicity backgrounds.
Data exclusions	No data were excluded from the analysis.
Replication	The three validation cohorts composed of the SG10K Project with 4,810 genomes on East Asian and South Asian, the ChinaMAP project with 10,588 Chinese genomes, and the WBBC pilot project with 4,480 Chinese genomes.
Randomization	Not relevant to this study. Variant allele frequencies were based on aggregated results.
Blinding	Sequencing data were collected by researchers from the gnomAD, SG10K, ChinaMAP and WBBC database, respectively. Authors from this study were not involved in sample acquisition of these studies.

Reporting for specific materials, systems and methods

We require information from authors about some types of materials, experimental systems and methods used in many studies. Here, indicate whether each material, system or method listed is relevant to your study. If you are not sure if a list item applies to your research, read the appropriate section before selecting a response.

Materials & experimental systems

n/a	Involved in the study
☒	☐ Antibodies
☒	☐ Eukaryotic cell lines
☒	☐ Palaeontology and archaeology
☒	☐ Animals and other organisms
☒	☐ Clinical data
☒	☐ Dual use research of concern

Methods

n/a	Involved in the study
☒	☐ ChIP-seq
☒	☐ Flow cytometry
☒	☐ MRI-based neuroimaging
